# The Principles and Practice of Dental Surgery

**Published:** 1850-09

**Authors:** 


### BIBLIOGRAPHICAL NOTICES.

*The Principles and Practice of Dental Surgery.* By CHAPIN A. HARRIS, M. D., D. D. S, Professor of the Principles and Practice of Dental Surgery in the Baltimore College, &c. &c. *Fourth Edition; revised, modified and greatly enlarged, with two hundred illustrations.* Philadelphia. Lindsay & Blakiston: 1850.

We hail with pleasure every advance made in this kindred branch of surgery, and gladly award the meed of praise to him who adds but a mite to the treasury of fast-accumulating lore in this department. Among the most energetic in the good cause of reform is the industrious author of the work before us, whose high appreciation by his brethren is manifested in the rapid sale of three large editions and the appearance of a fourth. The days of empiricism and charlatanism are fast disappearing under the labors of the author and his colleagues, and other colaborers, and scientific attainments are rapidly becoming a sine qua non to him who would hope for even moderate success in his profession. Mere mechanical dexterity is not all that is necessary to make an accomplished dentist. A knowledge of Anatomy, Physiology, Hygiene and General Therapeutics, are even more essential to conservative dentistry, the great end and aim of every judicious practitioner.

These general remarks have been drawn forth by the perusal of the volume before us, which although familiar to us, and doubtless to the mass of the profession, from an acquaintance with former editions, still demands that we should speak in flattering terms of the care and erudition displayed in its production. We observe three new chapters on "Filling Teeth" and one on "Mechanical Dentistry," with the addition of forty-five new engravings. In mechanical execution the work cannot be excelled, and we predict for the present edition an equally favorable reception as those of its predecessors.

